# Rehabilitation and Return to Sport Assessment after Anterior Cruciate Ligament Injury: Quantifying Joint Kinematics during Complex High-Speed Tasks through Wearable Sensors

**DOI:** 10.3390/s21072331

**Published:** 2021-03-26

**Authors:** Stefano Di Paolo, Nicola Francesco Lopomo, Francesco Della Villa, Gabriele Paolini, Giulio Figari, Laura Bragonzoni, Alberto Grassi, Stefano Zaffagnini

**Affiliations:** 1 Department for Life Quality Studies, University of Bologna, 40136 Bologna, Italy; stefano.dipaolo@ior.it (S.D.P.); laura.bragonzoni4@unibo.it (L.B.); 2 Department of Information Engineering, University of Brescia, 25123 Brescia, Italy; nicola.lopomo@unibs.it; 3 Isokinetic Medical Group, Educational & Research Department, FIFA Medical Centre of Excellence, 40132 Bologna, Italy; f.dellavilla@isokinetic.com; 4 GPEM srl, 07041 Alghero, Italy; gabriele.paolini@gpem.net (G.P.); giulio.figari@gpem.net (G.F.); 5 Orthopaedic and Traumatologic Clinic II, IRCCS Istituto Ortopedico Rizzoli, 40136 Bologna, Italy; alberto.grassi@ior.it; 6 Department of Biomedical and Neuromotor Sciences, University of Bologna, 40136 Bologna, Italy

**Keywords:** wearable inertial sensors, marker-based optoelectronic system, ACL, rehabilitation, motion capture validation, kinematics

## Abstract

The aim of the present study was to quantify joint kinematics through a wearable sensor system in multidirectional high-speed complex movements used in a protocol for rehabilitation and return to sport assessment after Anterior Cruciate Ligament (ACL) injury, and to validate it against a gold standard optoelectronic marker-based system. Thirty-four healthy athletes were evaluated through a full-body wearable sensor (MTw Awinda, Xsens) and a marker-based optoelectronic (Vicon Nexus, Vicon) system during the execution of three tasks: drop jump, forward sprint, and 90° change of direction. Clinically relevant joint angles of lower limbs and trunk were compared through Pearson’s correlation coefficient (r), and the Coefficient of Multiple Correlation (CMC). An excellent agreement (r > 0.94, CMC > 0.96) was found for knee and hip sagittal plane kinematics in all the movements. A fair-to-excellent agreement was found for frontal (r 0.55–0.96, CMC 0.63–0.96) and transverse (r 0.45–0.84, CMC 0.59–0.90) plane kinematics. Movement complexity slightly affected the agreement between the systems. The system based on wearable sensors showed fair-to-excellent concurrent validity in the evaluation of the specific joint parameters commonly used in rehabilitation and return to sport assessment after ACL injury for complex movements. The ACL professionals could benefit from full-body wearable technology in the on-field rehabilitation of athletes.

## 1. Introduction

Biomechanical assessment of human movement represents a key tool to discriminate normal and pathological patterns in a wide variety of applications. In the sport-related context, the risky patterns lead to an increased risk for severe injuries such as the non-contact Anterior Cruciate Ligament (ACL) injury [1,2,3,4,5]. The ACL injury has detrimental consequences: a return to sport (RTS) at pre-injury level is not guaranteed, the re-injury rate is high (up to 30% [6,7]), and the risk of post-traumatic osteoarthritis increases by 4-fold [8]. All the main joints have a role in the ACL injury mechanism [1,9,10], and targeted neuromuscular training has been proposed to modify the specific risky patterns in the rehabilitation phase after injury [11,12,13,14]. Thus, the interest in tools for assessing multi-joint biomechanics in this context has increased more and more [15,16]. Such evaluation could help to understand the injury risk in high-speed and multidirectional movements, thus supporting the rehabilitation phase.

The optoelectronic marker-based (OMB) motion capture approach represents the gold standard for biomechanical evaluations. The OMB systems have been used for multiple applications in the analysis of both healthy and disease-related movement patterns [4,17]. Nevertheless, when it comes to an ACL scenario, the main and well documented-limitations of this technology are amplified: there is a need for a dedicated space, causing difficult on-field applications; there is a high cost in terms of money, time and technical skills; it is difficult to obtain quick reports [18,19]. The alternative solution is represented by wearable inertial sensors (WIS). This technology is indeed portable, less cumbersome on the body, and can produce real-time results. Different uses of the WIS technologies have been reported: single sensors settings have been mainly used in the assessment of athletes’ performances at both individual and team level; multiple sensors settings have been mainly used for biomechanical assessments of both healthy movement patterns and to identify the effects of several diseases, including different contexts and applications [18,19,20].

The advantages brought by the WIS systems might have a strong impact on the ACL rehabilitation; ACL professionals might indeed benefit from a direct assessment of the biomechanical predictors of ACL injury risk, while performing the evaluation in players’ actual environments, thus reducing the bias caused by controlled conditions and limited space.

Although previous studies have been carried out to validate the WIS against the OMB, demonstrating consistent results and good accuracy [18], extensive use of the WIS technology in the assessment of athletes’ biomechanics during ACL rehabilitation is still not reported. A possible explanation is that no validation of WIS has been performed on clinically relevant parameters addressing the analysis of ACL injury risk in complex movements; in fact, movement complexity plays a significant role in motion capture system accuracy, adding extra sources of noise on the expected outcomes [19,21]. A feasibility analysis on clinically relevant parameters for ACL professionals is also crucial to focus on modifiable risk factors and athletes’ progress. At present, little knowledge exists on the reliability of the WIS systems with respect to such specific requirements.

Therefore, the aim of the present study was to quantify joint kinematics through a full-body WIS system and to test its concurrent validity against a gold standard OMB system in a set of multidirectional high-speed complex movements included in a protocol for rehabilitation and RTS assessment after ACL reconstruction.

The main hypothesis was that the WIS system could be reliably used in the analysis of multi-joint kinematics when focusing on clinically relevant parameters and tasks.

## 2. Materials and Methods

The experimental session was conducted in the Green Room of the Isokinetic Medical Center of Bologna (Italy). An a priori power analysis was performed based on a previous similar study [22] to determine the correct sample size. At least 28 athletes were required to have a power of 0.9 with a minimum effect size of 0.5 (large) and a type I error of 0.05.

Overall, 34 recreational and elite athletes were recruited for the study (Table 1). Inclusion criteria were that they were aged between 18–50 years old, and had a Tegner level of at least 5. Exclusion criteria were musculoskeletal disorders or impairment, BMI > 35, previous surgery to lower limbs, and cardiopulmonary or cardiovascular disorders.

All the athletes signed informed consent before starting the acquisition protocol. The research study was approved by the Institutional Review Board (IRB approval: 555/2018/Sper/IOR of 12/09/2018) of Area Vasta Emilia Romagna Centro (AVEC, Bologna, Italy) and registered on ClinicalTrials.gov (Identifier: NCT03840551) (accessda on 15 march 2021).

### 2.1. Experimental Protocol

The analysis was performed in a specialized laboratory, equipped with artificial turf. Regarding the OMB motion capture, a set of 10 stereophotogrammetric cameras (Vicon Vero, Vicon Motion Systems Ltd., Oxford, UK) with a sampling frequency of 120 Hz were used. Three synchronized, calibrated, high-speed RGB cameras were also used to record the movement. The calibration of cameras and volume of the acquisition was performed at the beginning of the acquisition and repeated periodically during the session. A total of 42 retroreflective markers were placed on each athlete according to the full-body Plug-in Gait protocol (Appendix A, top row).

Regarding the WIS system, a set of 15 inertial sensors (MTw Awinda, Xsens Technologies, Enschede, Netherlands) with a sample frequency of 60 Hz were placed according to the Full body No Hands Xsens protocol (Appendix A, bottom row). The WIS has an internal sampling rate of 1000 Hz, an accelerometer range of 16 g, a gyroscope range of ± 2000°/s, and a dynamic systematic uncertainty of 0.75°.

During data acquisition, the athletes were contemporary equipped with both systems. A single operator (G.P.) carried out the athletes’ sensor and marker placement. After marker and sensor positioning, both static and dynamic subject calibrations were performed simultaneously for the two systems, and anthropometric measurements were collected. Data capture was triggered via hardware by using the OMB system as a master and the WIS system as a slave to directly compare the two systems’ acquisitions.

**Figure 1:**
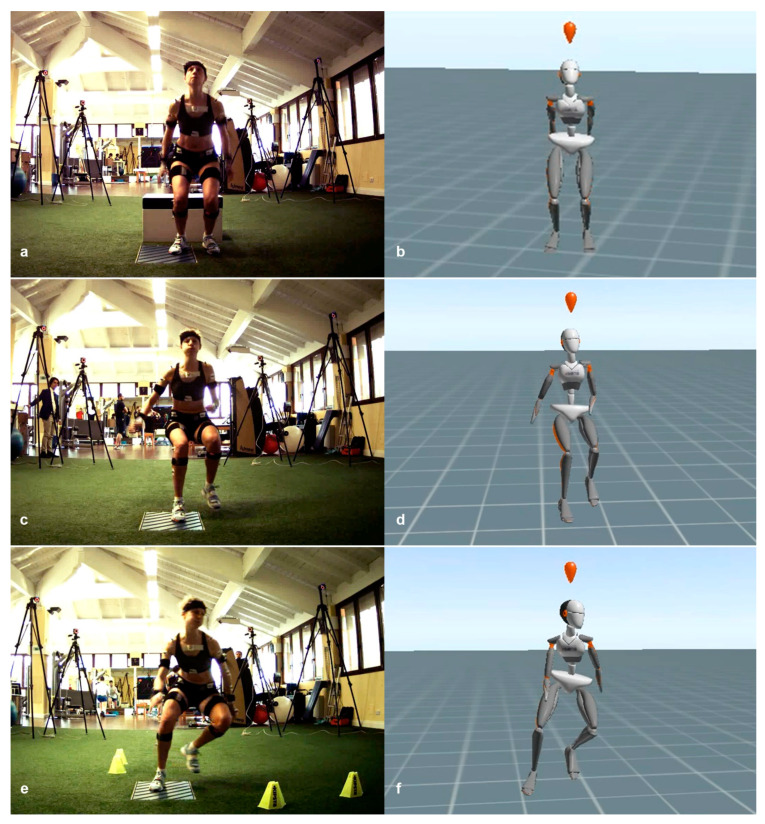
Representation of the three motor tasks performed by the athletes and used for comparative analysis alongside the real-time movement reconstruction in the wearable sensor software environment: (**a**,**b**) the drop jump (DJ); (**c**,**d**) the forward sprint (FS); (**e**,**f**) the change of direction at 90° (CD).

Each athlete performed three motor tasks: a drop jump (DJ), a forward sprint (FS), and a change of direction at 90° (CD). The DJ consisted of a bilateral landing from a 41 cm-high box, with an immediate vertical jump at maximum force [2]. The FS consisted of a frontal sprint followed by a sudden stop on a single leg and by a further backward sprint, all performed at maximum speed [23]. The CD consisted of a frontal sprint followed by a sudden sidestep cutting maneuver at 90° and a further frontal sprint in the new direction, all performed at maximum speed [24] (Figure 1). These motor tasks are included in a protocol for the biomechanical assessment of return to sport after ACL reconstruction, developed and currently deployed at the Isokinetic Medical Group, a FIFA Medical Centre of Excellence.

Before the real test, the athletes were instructed and performed a few warm-up repetitions of each task in order to get confident with the movement. A sports medicine physician (F.D.V.) instructed each athlete on the movements performed and verified each trial’s validity. All the athletes performed three valid repetitions of each task per leg (18 total valid trials per athlete). The tasks were performed consecutively, after a short rest (of a few seconds) per each athletes’ fatigue.

### 2.2. Features Selection and Data Processing

**Figure 2:**
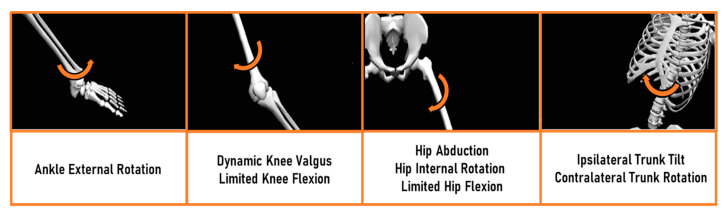
Common ACL injury mechanism according to the current literature. From the left: ankle, knee, hip, and trunk joint main kinematical mechanisms found in ACL injury. Arrows indicate the direction of joint motion. The limitation of these patterns is the goal of targeted neuromuscular training in rehabilitation after ACL injury. The figure is adapted from Della Villa et al. [1].

The kinematic parameters’ selection was based on the current concepts of ACL injury mechanisms in the orthopedics and sports medicine community [1,2,3,11]. Since the main injury pattern, and the neuromuscular training to prevent it, involves both the lower limbs and the trunk complex, the ankle, knee, hip, and trunk joint angles were taken into account. Although a complete kinematical comparison (frontal, transverse, and sagittal plane for all the joints) between the systems was performed, the final analysis was focused on the following parameters: ankle transverse plane, knee frontal and sagittal plane, hip frontal, transverse and sagittal plane, trunk frontal and transverse plane (Figure 2). Data were resampled and normalized by considering the overall time length of each trial (0-100% of the task) to perform a direct comparison of each data frame. Joint angles were verified to match common conventions in all the three anatomical planes: abduction (+), adduction (-); internal (+), external (-) rotation; flexion (+), extension (-) [22].

### 2.3. Statistical Analysis

The normal distribution of the data was verified through the Shapiro-Wilk test. Data were compared using different metrics [17,18,25,26]: Pearson’s correlation coefficient (r); the Coefficient of Multiple Correlation (CMC); the offset defined as the difference between the means of the waveforms (ΔOFF); the Normalized Root Mean Square Error (NRMSE).

Pearson’s r and the CMC coefficient indicated the agreement between the systems. For Pearson’s r, the statistical significance of the correlation was also assessed with an alpha level of 5%. The CMC, calculated according to the definition given in Ferrari et al. [25], defined the similarity across the full movement, after offset removal. The validity was considered excellent if r and CMC > 0.75, fair if r and CMC 0.4–0.74, and poor if r and CMC < 0.39 [27].

The average offset error indicated the systematic error between the two waveforms and was expressed in degrees. In order to keep the information regarding which was the highest value between the two systems, positive values of ΔOFF indicated higher values for the OMB system, while negative values indicated higher values for the WIS system. The agreement was considered excellent for values of ΔOFF lower than ±5° [28,29]. The NRMSE indicated the dispersion of the data along with the waveforms and was normalized over the range of motion of the OMB system, thus ranging from 0 to 1. The validity was considered excellent for NRMSE < 0.2 [19]. All the statistical analyses were performed in MATLAB (The MathWorks, Natick, MA, USA).

## 3. Results

The athletes’ age was 22.8 ± 4.1 years, and the Tegner level was 8.6 ± 1.0 (Table 1). Overall, a total of 469 valid trials (77% of the total)—168 for DJ, 137 for FS, 164 for CD—were kept and compared between the two systems. Trial exclusion was made due to technical reasons, i.e., error in data acquisition or export of either the OMB or the WIS system data. The waveforms for all the kinematical parameters investigated can be found in Appendix B.

### 3.1. Drop Jump

Excellent agreement was found for the knee and hip sagittal plane angles (r = 0.99, CMC = 0.98) (Table 2). A fair-to-excellent agreement was found for knee, hip, and trunk frontal plane angles (r 0.69–0.81, CMC 0.67–0.88). Fair agreement was found for ankle, hip, and trunk transverse plane angles (r 0.58–0.74, CMC 0.63–0.83). Errors (ΔOFF) were always lower than 5°, except for hip sagittal plane angles (–6.91°, NRMSE = 0.1, Table 3). Higher average values on lower limb frontal and transverse plane angles were found for the OMB system at the peak flexion angles (Appendix B).

### 3.2. Frontal Sprint

Excellent agreement was found for the knee and hip sagittal plane angles (r > 0.94, CMC = 0.98) (Table 2). A fair-to-excellent agreement was found for frontal and transverse plane joint angles (r 0.47–0.85, CMC 0.59–0.91). The ΔOFF were lower than 1° and 7° for trunk and hip angles, respectively (Table 3). The highest offset errors were found for knee frontal (−9.93°, NRMSE = 0.43) plane angles, with higher average values for the OMB system (Appendix B).

### 3.3. 90° Change of Direction

Excellent agreement was found for the knee and hip sagittal plane angles, and for hip and trunk frontal plane angles (r > 0.87, CMC > 0.91) (Table 2). A fair-to-excellent agreement was found for the remaining frontal and transverse plane joint angles (r 0.45–0.87, CMC 0.66–0.91). The ΔOFF were always lower than 6°, except for knee frontal and ankle transverse plane angles (Table 3). The OMB system showed higher average values for knee frontal and transverse angles (Appendix B).

## 4. Discussion

The aim of the present study was to quantify joint kinematics and validate a full-body WIS motion capture system against a gold standard OMB in complex movements specifically used for the clinical evaluation of the ACL injury risk and return to sport. The analysis was carried out on a consistent number of healthy athletes, mainly coming from competitive sports (Tegner level 9 in 85% of athletes).

The main finding of the present study was that the WIS motion capture system showed overall fair-to-excellent correlation with respect to the OMB system, and acceptable measurement errors in all the movements assessed. This finding confirmed the concurrent validity of the WIS system already underlined in previous studies [18,28,29,30], and further extended its usability to clinical applications in the ACL rehabilitation context. For the first time, a stronger focus was put on clinically relevant biomechanical parameters used by the ACL professionals in the rehabilitation protocols after ACL injury in sport-specific, high-speed, and multidirectional movements. Indeed, the parameters investigated are the common targets of neuromuscular training used by sports physicians and orthopedic surgeons in the rehabilitation phase, and to clear patients for RTS [12,13,16,31,32,33] (Figure 2). The latter finding represents a great step forward in the use of full-body wearable technology by health professionals for ACL rehabilitation and RTS, both in-lab and on-field.

The differences found between the two systems were tolerable according to the literature requirements [18,19,27,28,29]. Knee and hip sagittal plane angles showed the highest agreement between the two systems (minimum CMC = 0.95). Similar levels of agreement and errors were reported in literature considering the very same WIS system used in the present study and either the same [22] or different OMB systems [28,29,34] addressing walking, stair climbing, and landing tasks. The results of the present study confirmed that sagittal plane angles could be accurately assessed in sprints and counter-movements. A trustful evaluation of sagittal plane joint angles is of primary importance for ACL injury risk and RTS. Many rehabilitation programs focus on reaching a good joint range of motion in dynamic tasks, as this reduces the stress on lower limb joints and the trunk [5,35,36]. Landing strategies favoring the hip (i.e., higher hip than knee flexion, namely “hip strategy”) or knee (i.e., higher knee than hip flexion, namely “knee strategy”) are also widely assessed in rehabilitation programs since they were shown to correlate with lower and higher knee abduction moment, respectively [37].

The agreement between the two systems on the frontal plane was fair-to-excellent for the hip and trunk, and fair for the knee. Measurement errors and agreement were only slightly affected by the different complexity of the three movements evaluated. This aspect partially extended the validity of the WIS system on frontal plane angles to include complex movements. Furthermore, the average and range of motion data were generally higher for the OMB system compared to the WIS system, particularly for the knee joint. These findings are in line with previous studies which focused on either gait or counter-movements [22,38]. The evaluation of frontal plane angles is crucial in the assessment of ACL injury risk and RTS [39,40]. Primary attention is paid to knee and hip frontal plane kinematics. The limitation of the dynamic valgus pattern represents a milestone of every ACL rehabilitation program since literature extensively underlines how this pattern increases the knee abduction moment and it is present in almost all the ACL rupture mechanisms in the athletes [1,2]. Trunk kinematics is also largely evaluated since excessive homolateral lean also increases knee abduction moment [41].

Fair-to-excellent agreement was found for the hip and trunk transverse plane angles. This aspect extends the possibilities of the ACL professionals during the rehabilitation: without 3D technologies, the correct assessment of such angles is critical and often neglected. The importance of rotational patterns has been already highlighted: the coupling of hip abduction and internal rotation leads to the dynamic knee valgus [4,24,42,43], and the excessive trunk rotation magnifies the knee’s external moments and the loss of core stability [41,42,44]. The lowest agreement was found for ankle transverse plane angles. The errors also increased with movement complexity (highest for the DJ task, lowest for the CD task). As for the knee frontal plane angles, absolute average values and range of motion were higher for the OMB system compared to the WIS in all the tasks. All the previous studies investigating the validity of WIS systems against the OMB systems reported a low agreement for transverse plane kinematics [17,18,20,28,29,37]. The assessment of such angles is critically related to the definition of the biomechanical models, the markers/sensors’ positioning, and the limited range of motion [18,28]. Ankle joint definition is probably the most critical for both the OMB and the WIS systems [26,45]. For the former, the relatively large shape of medial and lateral malleoli introduces complexity in landmark palpation [28] and the presence of an expert operator is mandatory. For the latter, the malleoli are automatically placed at the same height in the biomechanical model, thus probably introducing an offset on frontal orientation with respect to the OMB reference system. Moreover, the limited range of motion of such angles has been associated with an intrinsic decrease of measurement agreement like the CMC [46].

Regarding the clinical interpretation of the results obtained, the high peaks and range of motion evaluated through OMB seem to be highly non-physiological, even for counter-movements. This aspect was likely due to cross-talk between the sagittal and frontal plane in the OMB data analysis, thus mainly related to marker placement. Although this aspect could be of limited interest in gait analysis, it could cause severe flaws in an ACL scenario, where such high varus/valgus and internal/external rotation values could cause unreasonable alerts in the data interpretation [26]. The same joint angles evaluated with the WIS systems are much smaller. Despite this could be symptomatic of an “over-constrained” biomechanical model, these results seem more appropriate for a healthy athlete’ population. In clinical scenarios, attention should be paid when interpreting specific angle values. The strength of such WIS system relies on the kinematical assessment of multiple joints, which offers an overall consideration of the movement. ACL professionals should, therefore, take into account multiple variables when drawing clinical conclusions from such analyses.

The novelty of the present work relies on the clinically-oriented analysis carried out in terms of both motor tasks and parameters evaluated. Compared to the previous literature, the set of motor tasks evaluated in the present study is one of the most demanding in terms of complexity and has, most of all, practical clinical use in rehabilitation after ACL injury and the RTS in terms of parameters assessed. Therefore, the WIS system used in the present study (MTw Awinda, Xsens) resulted in a suitable solution for motion capture in the sports environment for the biomechanical assessment of ACL rehabilitation. The assessment of wearables’ accuracy before experimental applications has been recently advocated in specific applications such as outdoor sport and the military [19,47]. The present study, alongside all the limitations of the OMB system in outdoor use, endorses the use of WIS in ACL rehabilitation during on-field assessments. Such kinematical analyses could be of crucial importance to deeply evaluate movement quality directly on-field, thus improving and personalizing the rehabilitation strategies [20,48,49,50].

The present study has some limitations. First, the athletes’ velocity was not controlled. Although each athlete performed the movement at his/her best, intra-subject differences could have influenced the obtained outcome. Such analysis could contribute to understanding whether differences between the two systems increase over a defined level of velocity. Second, the systems’ comparison was based only on healthy athletes’ kinematic data. The analysis of those with ACL injuries or ACL reconstructed athletes could offer stronger insights on the sensibility of the two systems and should be objectives of future investigations. Third, the systems’ comparison was based on kinematic data only. Recent studies underlined the correlation between the angular velocity evaluated through WIS and the knee abduction moment evaluated through OMB and force platform in single-leg landing [51]. The assessment of joint moments, powers and inter-segmental forces is an intrinsic limitation of the wearable technology compared to optical tracking, alongside a lower applicability in specific motion capture fields, i.e., facial, hands, etc. Furthermore, the accuracy of the WIS system on knee and ankle frontal and transverse plane angles was flawed by the high—and probably non-physiological—values of the OMB systems. This is reported in the literature as an intrinsic limitation of the OMB biomechanical model and protocol selected for the study, which, on the other hand, were selected for their ease of use [18,47]. Lastly, a single full-body configuration was adopted for the WIS system. The possibility to reduce the number of inertial units while achieving a comparable accuracy [52] should be an objective of future investigations.

## 5. Conclusions

The full-body WIS motion capture system showed a fair-to-excellent concurrent validity in the evaluation of complex movements commonly used in rehabilitation after ACL injury. The ACL professionals could benefit from full-body wearable technology in the on-field rehabilitation of athletes.

## Figures and Tables

**Table 1:** Demographic data, mean ± SD [range].

Number of athletes	34
Age (years)	22.8 ± 4.1 [18,19,20,21,22,23,24,25,26,27,28,29,30,31]
Gender (m/f)	18/16
Body Mass Index	22.6 ± 2.6 [18,19,20,21,22,23,24,25,26,27]
Dominant limb (r/l) ^1^	30/4
Tegner	8.6 ± 1.0 [5,6,7,8,9]

^1^ Dominant limb is meant as the preferred one used to kick a ball.

**Table 2:** Agreement measurements (r, CMC) between the two motion capture systems for the three motor tasks, mean [range].

	r			CMC		
	DJ	FS	CD	DJ	FS	CD
**Ankle**						
Transverse	0.58 [0.49, 0.67]	0.53 [0.43, 0.62]	0.45 [0.35, 0.55]	0.63 [0.57, 0.69]	0.63 [0.56, 0.69]	0.62 [0.55, 0.69]
**Knee**						
Frontal	0.69 [0.59, 0.79]	0.55 [0.43, 0.67]	0.60 [0.50, 0.70]	0.67 [0.58, 0.76]	0.65 [0.57, 0.73]	0.63 [0.55, 0.72]
Sagittal	0.99 [0.98, 0.99]	0.95 [0.94, 0.97]	0.95 [0.93, 0.96]	0.99 [0.99, 0.99]	0.98 [0.97, 0.98]	0.97 [0.96, 0.98]
**Hip**						
Frontal	0.81 [0.76, 0.86]	0.85 [0.81, 0.89]	0.96 [0.95, 0.97]	0.88 [0.85, 0.91]	0.91 [0.88, 0.93]	0.96 [0.96, 0.97]
Transverse	0.64 [0.55, 0.73]	0.47 [0.34, 0.59]	0.63 [0.56, 0.71]	0.74 [0.68, 0.81]	0.59 [0.48, 0.70]	0.72 [0.67, 0.77]
Sagittal	0.99 [0.98, 0.99]	0.97 [0.97, 0.98]	0.96 [0.94, 0.97]	0.99 [0.99, 0.99]	0.98 [0.98, 0.99]	0.97 [0.96, 0.97]
**Trunk**						
Frontal	0.77 [0.72, 0.82]	0.67 [0.55, 0.79]	0.87 [0.84, 0.90]	0.85 [0.82, 0.89]	0.81 [0.75, 0.88]	0.91 [0.89, 0.94]
Transverse	0.74 [0.67, 0.81]	0.84 [0.79, 0.89]	0.81 [0.76, 0.86]	0.83 [0.78, 0.88]	0.90 [0.87, 0.93]	0.89 [0.87, 0.92]

Note: DJ = Drop Jump; FS = Frontal Sprint; CD = Change of Direction at 90°; CMC = Coefficient of Multiple Correlation.

**Table 3:** Error measurements (ΔOFF, NRMSE) between the two motion capture systems for the three motor tasks, mean [range].

	ΔOFF (°)			NRMSE (%)		
	DJ	FS	CD	DJ	FS	CD
**Ankle**						
Transverse	3.31 [0.38, 6.24]	−8.51 [−11.39, −5.64]	−7.91 [−11.16, −4.66]	0.26 [0.23, 0.28]	0.31 [0.27, 0.35]	0.37 [0.32, 0.42]
**Knee**						
Frontal	−4.14 [−5.90, −2.37]	−9.93 [−13.63, −6.22]	−10.93 [−14.67, −7.19]	0.27 [0.22, 0.32]	0.43 [0.35, 0.51]	0.40 [0.33, 0.47]
Sagittal	−4.67 [−6.63, −2.71]	−2.45 [−5.24, 0.35]	−3.86 [−6.28, −1.43]	0.08 [0.06, 0.09]	0.12 [0.09, 0.14]	0.12 [0.10, 0.14]
**Hip**						
Frontal	3.91 [2.55, 5.27]	4.82 [3.19, 6.45]	5.18 [3.38, 6.99]	0.36 [0.28, 0.44]	0.29 [0.23, 0.35]	0.21 [0.17, 0.25]
Transverse	−1.05 [−4.69, 2.6]	6.57 [1.83, 11.31]	5.49 [1.21, 9.76]	0.26 [0.22, 0.29]	0.38 [0.31, 0.45]	0.30 [0.25, 0.35]
Sagittal	−6.91 [−9.13, −4.68]	−2.94 [−5.62, −0.27]	−4.99 [−7.54, −2.44]	0.10 [0.09, 0.12]	0.13 [0.10, 0.16]	0.14 [0.12, 0.17]
**Trunk**						
Frontal	−0.33 [−1.30, 0.64]	−0.69 [−2.0, 0.61]	−1.05 [−2.40, 0.31]	0.30 [0.25, 0.35]	0.31 [0.25, 0.37]	0.20 [0.17, 0.23]
Transverse	0.38 [−1.06, 1.83]	−0.85 [−2.66, 0.97]	−1.14 [−3.26, 0.98]	0.37 [0.30, 0.45]	0.21 [0.17, 0.25]	0.26 [0.21, 0.30]

Note: DJ = Drop Jump; FS = Frontal Sprint; CD = Change of Direction at 90°; ΔOFF = difference between means of the waveforms; NRMSE = Normalized Root Mean Square Error.

**Figure A1:**
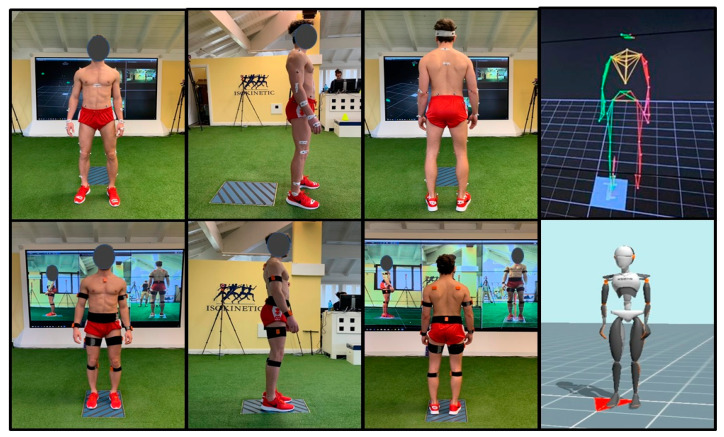
Motion capture systems used in the study: (top row) Plug-in-Gait VICON marker-based protocol in (from left to right) front, side, back, and 3D-environment view; (bottom row) Full Body Xsens inertial sensors protocol in (from left to right) front, side, back, and 3D-environment view.

**Figure A2:**
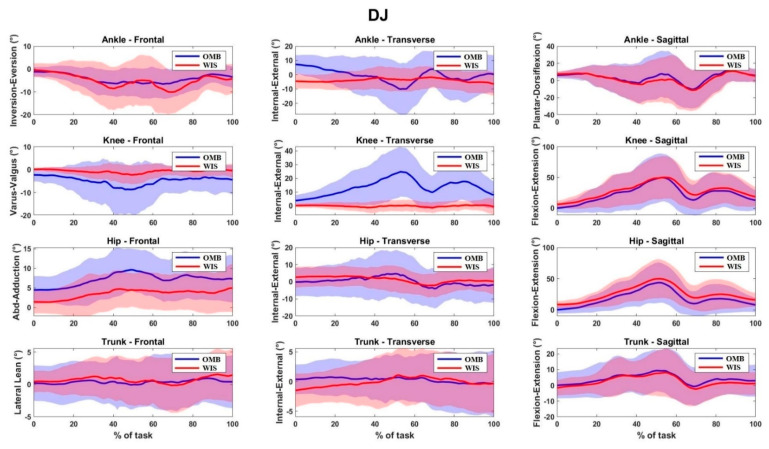
Joint kinematics of marker-based (OMB, blue) and inertial sensors (WIS, red) for the drop jump (DJ) task. Data are normalized by the length of the motor task. The waveforms are shown as average (solid lines) and standard deviations (shaded lines).

**Figure A3:**
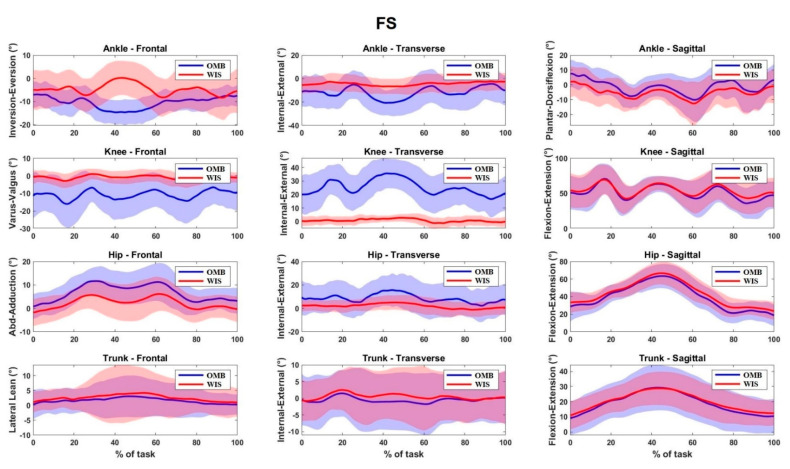
Joint kinematics of marker-based (OMB, blue) and inertial sensors (WIS, red) for the forward sprint (FS) task. Data are normalized by the length of the motor task. The waveforms are shown as average (solid lines) and standard deviations (shaded lines).

**Figure A4:**
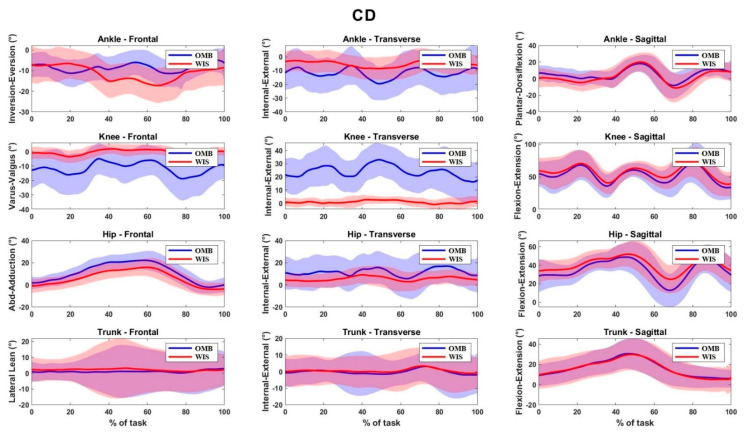
Joint kinematics of marker-based (OMB, blue) and inertial sensors (WIS, red) for the change of direction (CD) task. Data are normalized by the length of the motor task. The waveforms are shown as average (solid lines) and standard deviations (shaded lines).

## Data Availability

Data are available on reasonable request and due to restrictions, e.g., privacy or ethical.
